# Suppression of HIV-1 replication by microRNA effectors

**DOI:** 10.1186/1742-4690-6-26

**Published:** 2009-03-09

**Authors:** Christine Chable-Bessia, Oussama Meziane, Daniel Latreille, Robinson Triboulet, Alessia Zamborlini, Alexandre Wagschal, Jean-Marc Jacquet, Jacques Reynes, Yves Levy, Ali Saib, Yamina Bennasser, Monsef Benkirane

**Affiliations:** 1 Institut de Génétique Humaine CNRS UPR1142, Laboratoire de Virologie Moléculaire, Montpellier, France; 2 CNRS UMR7151 et Conservatoire National des Arts et Métiers, Paris, France; 3 Service des Maladies Infectieuses et Tropicales, Centre hospitalier universitaire Montpellier, France; 4 INSERM, Unite U841, Université Paris 12, Faculté de Médecine, AP-HP, Groupe Henri-Mondor Albert-Chenevier, Immunologie clinique, Créteil, F-94010, France

## Abstract

The rate of HIV-1 gene expression is a key step that determines the kinetics of virus spread and AIDS progression. Viral entry and gene expression were described to be the key determinants for cell permissiveness to HIV. Recent reports highlighted the involvement of miRNA in regulating HIV-1 replication post-transcriptionally. In this study we explored the role of cellular factors required for miRNA-mediated mRNA translational inhibition in regulating HIV-1 gene expression. Here we show that HIV-1 mRNAs associate and co-localize with components of the RNA Induced Silencing Complex (RISC), and we characterize some of the proteins required for miRNA-mediated silencing (miRNA effectors). RCK/p54, GW182, LSm-1 and XRN1 negatively regulate HIV-1 gene expression by preventing viral mRNA association with polysomes. Interestingly, knockdown of RCK/p54 or DGCR8 resulted in virus reactivation in PBMCs isolated from HIV infected patients treated with suppressive HAART.

## Background

RNA silencing (RNAi) is a new gene regulatory mechanism conserved from plants to humans. RNAi mediators are small non-coding RNAs (sncRNAs) that function through sequence specific mRNA targeting to either induce their degradation and/or inhibit translation [1,2]. In mammals, RNAi is mediated by different classes of small non-coding RNAs including piRNAs, microRNAs and siRNAs [3-5]. MicroRNAs are produced from a primary transcript (pri-miRNA) which is processed in the nucleus by the microprocessor complex containing RNase Drosha and DGCR8. The resulting product or pre-miRNA is exported to the cytoplasm through the exportin-5 pathway. Cytoplasmic pre-miRNA is processed by typeIII RNase Dicer to miRNA/miRNA* duplex of 19 to 25 nucleotides. miRNA/miRNA* is incorporated into the RNA-Induced Silencing Complex (RISC) where miRNA* is degraded while miRNA serves as a guide for mRNA targeting [2]. Key components of miRISC are proteins of the Argonaute family (Ago1 to Ago4) that are required for miRNA-mediated silencing [6,7]. To ensure mRNA translational inhibition and decay, miRISC, loaded with miRNA and its mRNA targets, associate with proteins involved in mRNA processing [2]. A key factor in this process is the GW182 protein that interacts directly with Argonaute1 (Ago1) [8], and the human homologs of GW182 that interact with Ago1–4 [9]. GW182 orchestrates both mRNA decapping, through the recruitment of p54/RCK that regulates the activity of the decapping enzymes DCP1/DCP2 [10], and mRNA deadenylation by recruiting the CCR4-NOT1 complex [11]. mRNA decapping and deadenylation leads to mRNA decay through the action of XRN1, a 5'-3' exonuclease [10]. Interestingly, RNAi effectors, including miRNAs and their target mRNAs, Ago proteins, GW182, RCK/p54, LSm-1 and DCP proteins co-localize in cytoplasmic structures called GW-bodies or P-bodies suggesting that miRNA-mediated silencing occurs at these sites [11-15]. Emerging evidence suggests that miRNA-mediated gene regulation serves as a defence mechanism against both RNA and DNA viruses in mammals [16-20]. The present study was designed to explore physical and functional interaction between effectors of miRNA-mediated silencing and HIV-1 replication.

## Results and discussion

**Figure 1:**
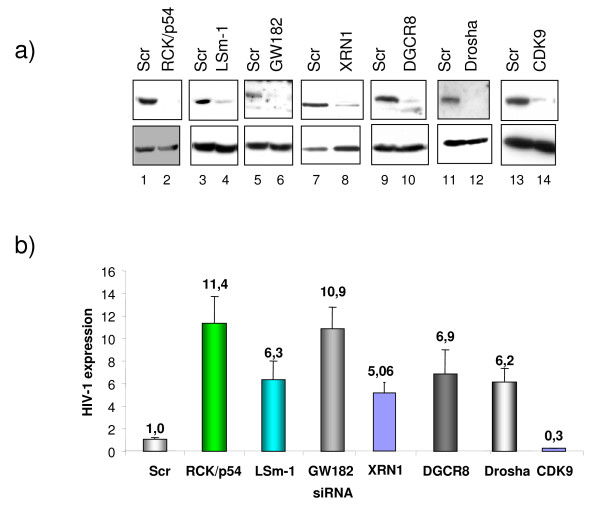
**miRNA effectors are repressors of HIV-1 replication**. HeLa cells were transfected with siRNA as indicated. 48 hours post transfection, cells were analyzed for RCK/p54, LSm-1, GW182, XRN1, DGCR8, DROSHA and CDK9 expression by Western blotting (a), or infected with a single round infectious virus (HIV-1-VSV-luc 200 ng/ml) and cell extracts were measured for luciferase activity 48 hours after infection (b). Results are presented as fold HIV production relative to Scr transfected cells, and data are representative of three independent experiments.

**Figure 2:**
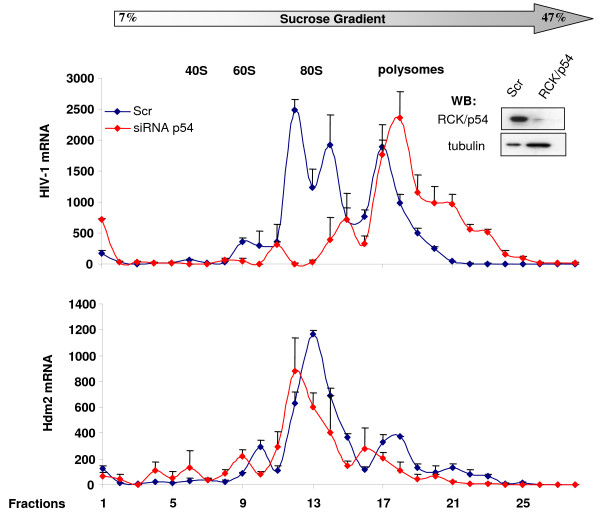
**RCK/p54 restricts HIV-1 mRNA association with polysomes**. Cytoplasmic extracts from HeLa cells that were transfected with the indicated siRNA and infected with HIV-1-VSVG-luc were run on glycerol gradient (7% to 47%). Fractions were collected and their RNA contents were monitored by measuring absorbance at 254 nm. HIV-1 mRNA (top panel) and Hdm2 mRNA (lower panel) were quantified in all the fractions by Q-RT-PCR using specific oligonucleotides.

To investigate whether RNAi effectors regulate HIV-1 replication, we analyzed virus replication in cells where expression of RNAi effectors was reduced using specific siRNA. HeLa cells were transfected with siRNA specific to RCK/p54, GW182, LSm-1 or XRN1. As controls, HeLa cells were transfected with scrambled siRNA (Scr) or CDK9 specific siRNA and subsequently infected with HIV-1 (Figure 1a). Knockdown of RCK/p54, GW182, LSm-1 and XRN1 enhanced virus replication by up to 10 fold (Figure 1b). As we have previously shown, knockdown of Drosha [21] and DGCR8 (Figure 1b), the two subunits of the microprocessor complex, increased virus production while knockdown of the CDK9 subunit of the PTEFb complex that is required for viral gene expression, reduced HIV-1 production (Figure 1b). Interestingly, analysis of HIV-1 cytoplasmic mRNA distribution on glycerol gradient showed that knockdown of RCK/p54 shifted HIV-1 mRNA from the non-polysomal fraction to polysomes as compared to control siRNA transfected cells (Figure 2, upper panel). As control, we analyzed the distribution of endogenous mRNA expressed from a gene encoding Hdm2. Knockdown of RCK/p54 did not affect Hdm2 mRNA distribution (Figure 2, lower panel). These experiments show that GW182, RCK/p54, LSm-1 and XRN1, factors required for RNAi, are repressors of HIV-1 gene expression that act by preventing HIV-1 mRNA translation.

**Figure 3:**
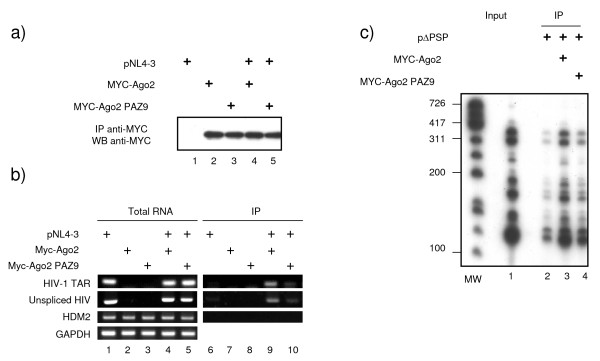
**HIV-1 mRNAs associate with Argonaute 2**. 293 cells were transfected with HIV-1 molecular clone pNL4-3, Myc-Ago2 or Myc-AgoPAZ9 as indicated. 48 hours later cells were harvested and cytoplasmic extracts were prepared. Total RNA was purified from a fraction of harvested cells while the rest was subjected to immunoprecipitation using anti-Myc antibody. After washing, a fraction was used to analyze the amount of Myc-Ago2 and Myc-Ago2PAZ9 immunoprecipitated by Western blotting (a), and the rest of the Myc-IPs was used for RNA extraction. HIV-1 mRNAs (TAR and unspliced), Hdm2 and GAPDH mRNA were quantified from total RNA (b, left panel) or from Myc immunoprecipitated mRNPs (b, right panel) by RT-PCR using specific oligonucleotides. c) Experiment was performed as in fig 3 except that 293 cells were transfected with HIV-1 ΔPSP which contains a partial gag/pol deletion but retains all the mRNA splicing sites [66], and 32P-labelled nucleotides were used in the PCR reaction. PCR products were visualized by autoradiography.

**Figure 4:**
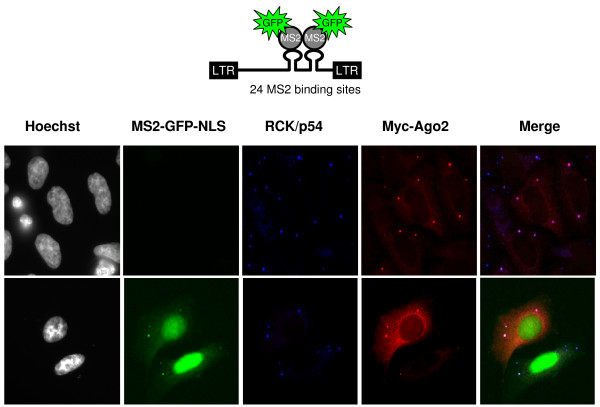
**HIV-1 mRNA co-localizes with RCK/p54 and Ago2**. HeLa cells were transfected with Myc-Ago2 expression vector either alone (top panels) or co-transfected with HIV-1 vector containing 24 repeats of MS2 binding sites and MS2-GFP expression vectors [64,65] (lower panels). Endogenous RCK/p54 and transfected Myc-Ago2 were visualized using specific primary antibodies and appropriate secondary antibodies coupled with Cy5 (shown in blue) and Cy3 (red) respectively. HIV-1 RNA bound to MS2-GFP is shown in green. Green, blue and red merged images are shown.

We next investigated the physical interaction between RNAi effectors and HIV-1 mRNA. 293 cells were mock transfected or transfected with combinations of pNL4-3, Myc-Ago2, a central component of the RISC complex, or its RNA-binding mutant Myc-Ago2PAZ9 constructs as indicated in figures 3 and 4. First, we verified that Myc-Ago2 and Myc-Ago2PAZ9 were equally expressed (Figure 3a). Second, cytoplasmic extracts were prepared, and a fraction was used for total RNA extraction while the rest was subjected to immunoprecipitation using anti-Myc antibody to purify Myc-Ago2 associated mRNP. Both total RNA (Figure 3b, left panels) and Myc-Ago2 associated RNA (Figure 3b, right panels) were reverse transcribed and subjected to PCR amplification using oligonucleotides specific for HIV-1 TAR RNA (a structured motif associated with all HIV-1 mRNAs) or HIV-1 unspliced mRNA, Hdm2 mRNA or GAPDH mRNA. PCR analysis of total RNA showed that equal amounts of HIV-1, Hdm2 and GAPDH mRNAs were present in all samples (Figure 3b, left panels). HIV-1 mRNAs (both TAR and unspliced) were associated with Myc-Ago2, but not with Myc-Ago2PAZ9 mutant (Figure 3b, right panels). In agreement with the results shown in figure 2, Hdm2 mRNA was not detected in Myc-Ago2 mRNPs, suggesting that under these conditions Hdm2 is not regulated by RNAi. A similar experiment was performed to analyze the association of HIV-1 multispliced mRNA with Myc-Ago2 mRNPs. The RT-PCR reactions were performed in the presence of 32P-α ATP and were analyzed by autoradiography (Figure 3c). HIV-1 multispliced mRNAs associated with Myc-Ago2 (compare lane 3 to 2) and weakly with Myc-Ago2PAZ9 (compare lane 4 to lanes 3 and 2). Co-localization of HIV-1 mRNA and effectors of RNAi such as Ago2 and RCK/p54 within the P-bodies was also observed by immunofluorescence using HIV-1 containing MS2 binding sites and MS2-GFP constructs (Figure 4). Indeed, HIV-1 mRNAs visualized through their binding to MS2-GFP colocalized with endogenous RCK/p54 and ectopically expressed Myc-Ago2 (Figure 4). Our results show that HIV-1 mRNAs physically associate with Ago2, a central component of RISC, and co-localize with cellular proteins required for miRNA-mediated silencing such as RCK/p54 and Ago2 in P-bodies. We observed that all HIV-1 mRNA species associated with RISC. Accordingly, Huang et al. had identified 5 cellular miRNAs able to target the 3'UTR sequence present in all HIV-1 mRNAs [22]. Additionally, other cellular miRNAs able to target regions out side the 3'UTR may also participate [23].

**Figure 5:**
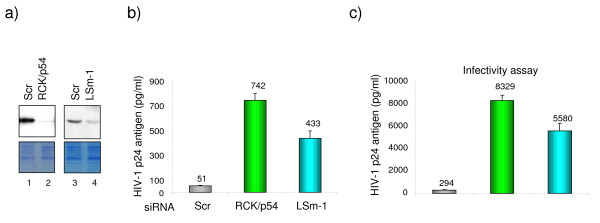
**Disruption of P-bodies through knockdown of RCK/p54 and LSm-1 leads to enhanced production of infectious HIV-1 virions**. HeLa CD4+ cells were transfected with siRNA as indicated. 48 hours post transfection cells were analyzed for RCK/p54 and LSm-1 expression by Western blotting (a) and infected with equal amounts of HIV-1 (200 ng/ml). b) Virus production was monitored 48 hours post infection by measuring p24 antigen in culture supernatant. c) To analyze the infectivity of new progeny virions, equal volumes of supernatant from siRNA transfected Hela CD4+cells were used to re-infect HeLa CD4+ cells. P24 antigen was measured in culture supernatant 48 hours post infection.

Emerging evidence suggests the physical and functional interactions between P-bodies and the viral life cycles [24]. Viral mRNA trafficking through P-bodies may represent a pool of translationally repressed viral transcripts otherwise used for efficient packaging or formation of viral-replication complexes. Indeed, yeast retrotransposons Ty1 and Ty3 mRNA associate with P-bodies, and this association is required for efficient retrotransposition [25-27]. In the case of BMV (Brome Mosaic Virus), formation of the virus replication complex occurs in P-bodies [28]. In addition, P-bodies may also function in host defences against viruses and transposable elements. Indeed, the cellular factors APOBEC 3G (A3G) and 3F (A3F), which are viral restriction factors, are found to accumulate in P-bodies [29,30]. It has been suggested that A3G and A3F mediated HIV-1 restriction may involve viral mRNA targeting to P-bodies leading to their translational inhibition [30]. We, therefore, asked whether P-bodies are positive or negative regulators of HIV-1 replication. Thus, we analyzed HIV-1 replication in cells where P-bodies were disrupted by knocking down RCK/p54 or LSm-1 [31]. HeLa CD4+ cells were transfected with RCK/p54 or LSm-1 specific siRNA or control siRNA. Forty eight hours later, cells were infected with equal amounts of HIV-1 viral particles (as measured by p24 assay). HIV-1 p24 antigen was measured in cell culture supernatant 48 hours post-infection. As shown in figure 5b, knockdown of RCK/p54 or LSm-1 results in enhanced virus production as compared to infection of control siRNA transfected cells. To assess the infectivity of the produced viruses, an equal volume of supernatant from Scr, RCK/p54 and LSm-1 siRNAtransfected cells was used to infect HeLa CD4+ cells, and p24 release in the culture supernatant was measured 48 hours later (Figure 5c). Virus infectivity correlated with the amount of p24 produced (Figure 5b) showing that virions produced in RCK/p54 and LSm-1 knocked down cells are fully competent for replication and have no defect in steps such as RNA packaging. Since the knockdown of RCK/p54 and LSm-1 was shown to result in the disruption of P-bodies, we concluded from these experiments that accumulation of HIV-1 mRNA in P-bodies limits virus replication.

**Figure 6:**
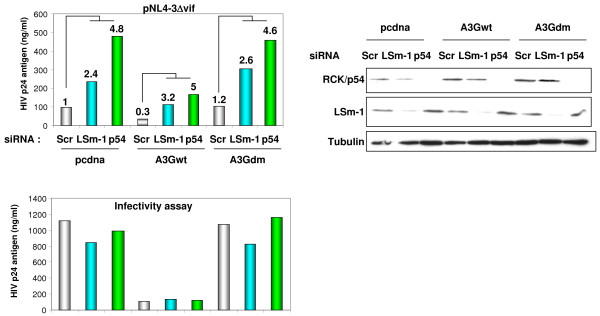
**RNAi effectors and APOBEC 3G-mediated HIV-1 repression involve different pathways**. HeLa CD4+ cells were transfected with the indicated siRNA. 48 hours later cells were analyzed for RCK/p54 and LSm-1 expression (right panel) or co-transfected with 1 μg of pNL4-3Δvif (lacking *vif *gene) and pcDNA or expression vectors for wild-type APOBEC3G or APOBEC3G double mutant lacking both deaminase and antiviral activity, A3G H65R/H257R [63]. HIV-1 production was measured 24 hours post-transfection in culture supernatant by quantifying p24 antigen (top left panel). Numbers on the top of the columns are fold increase relative to the respective Scr. Numbers on the top of Scr samples in A3Gwt and A3Gdm represent fold increase relative to Scr in pCDNA transfected cells. Infectivity assay was performed using equal amounts of p24 antigen to infect HeLa CD4+ cells. HIV-1 p24 antigen was measured 48 hours post infection (lower left panel). A representative experiment out of five is shown.

Next, we asked whether A3G-mediated HIV-1 restriction requires effectors of miRNA-mediated mRNA translational inhibition. Thus, we compared A3G-mediated HIV-1 restriction in cells where RCK/p54 or LSm-1 expression was reduced compared to control cells. HeLa cells were transfected with control siRNA or siRNA specific for RCK/p54 or LSm-1 (Figure 6, right panel). Forty-eight hours later, cells were transfected with an HIV-1 molecular clone lacking the *vif *gene (pNL4-3Δvif) either alone or with wild-type A3G or A3G mutant lacking antiviral activity (A3Gdm). HIV-1 p24 antigen was measured in culture supernatant 48 hours post-transfection. Interestingly, knock down of RCK/p54 or LSm-1 enhanced HIV-1 production regardless of A3G (Figure 6, left upper panel). Similarly, A3G but not A3Gdm reduced virus production regardless of RCK/p54 or LSm-1 expression (Figure 6, left upper panel). These results suggested that RCK/p54 or LSm-1 and A3G -mediated HIV-1 repression involves different mechanisms. We then analyzed the infectivity of HIV-1 produced from siRNA transfected cells. Equal amounts of p24 were used to infected HeLa CD4+ cells, and HIV-1 p24 antigen was measured in culture supernatant 48 hours post-infection. As shown in figure 6 (lower panel), virus produced in Scr siRNA transfected cells in the presence of A3G showed lower infectivity than those produced in its absence or in the presence of A3Gdm. Similar HIV-1-restriction activity of A3G was observed when the virus was produced in RCK/p54 or LSm-1 knocked down cells. This experiment showed that A3G-mediated HIV-1 restriction is independent of RNAi effectors RCK/p54 and LSm-1 and does not require P-bodies.

**Figure 7:**
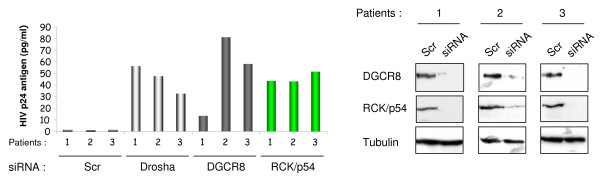
**Implication of RNAi in HIV-1 latency**. PBMCs were isolated from three patients undergoing active HAART. Isolated PBMCs were transfected with the indicated siRNA and either analyzed for RCK/p54, DGCR8 and DROSHA expression by Western blotting 48 hours after transfection (right panel) or co-cultured with activated PBMCs obtained from healthy donors. Virus replication was monitored every 3 to 4 days post co-culture by measuring p24 antigen in culture supernatant. Shown is the amount of p24 antigen at day 15 post co-culture. No virus was isolated from Scr transfected-PBMCs for up to 27 days.

Taken together, our results show a physically repressive interaction between RNAi effectors and HIV-1 mRNA. Since cellular miRNAs were shown to play a role in HIV-1 latency [22], we asked whether RCK/p54, which is required for miRNA-mediated mRNA translational inhibition, contributes to HIV-1 silencing *in vivo*. Thus, PBMCs isolated from 3 HAART-treated HIV-1-infected patients with undetectable viremia were transfected with control siRNA or with siRNA specific for Drosha, DGCR8 or RCK/p54. Transfected cells were co-cultured with PHA/IL2-activated PBMCs isolated from healthy donors. Virus production was monitored every 3 days by measuring p24 antigen in the culture supernatant (Figure 7). As we have previously shown, knockdown of Drosha resulted in virus reactivation in PBMCs isolated from 3 HAART-treated HIV-1-infected patients [21]. Remarkably, viral replication from its natural reservoir resumed also when DGCR8 or RCK/p54 was silenced. No virus was isolated from control siRNA transfected PBMCs suggesting that virus production observed in Drosha, DGCR8 and RCK/p54 knock down was not due to actively infected PBMCs relieved from drug pressure. These results show that endogenous levels of Drosha, DGCR8 and RCK/p54 contribute to HIV-1 latency and/or its maintenance in infected patients.

## Conclusion

The outcome of HIV-1 infection results from complex interactions between viral components and host cell factors [32-35]. In most cases, HIV-1 successfully hijacks cellular pathways and bypasses restriction factors for optimal replication leading to continuous rounds of infection, replication, and cell death. Continuous viral replication causes the loss of CD4+T cells and progression to immunodeficiency in infected individuals. HAART treatment revealed the existence of a pool of resting memory CD4+ T cells harbouring integrated, but silent HIV-1 provirus [36,37]. This latent reservoir is believed to be the major obstacle for virus eradication by HAART. Therefore, it is critical to understand how HIV-1 latency is established and maintained [38]. Post-integration latency takes place at both transcriptional and post-transcriptional levels [39]. Transcriptional latency involves different mechanisms ranging from integration position effect [40-42], limitation in transcription factors [43-46], establishment of chromatin repressive marks and recruitment of chromatin silencers [47-51]. Post-transcriptional silencing involves defects in mRNA export and translation [52-54]. All together, these studies show that HIV-1 post-integration latency is a multi-factorial process. In the present study, we show that HIV-1 gene expression is additionally regulated by the miRNA pathway. HIV-1 mRNA associates with components of the RISC complex by a mechanism that does not involve APOBEC3G, but does need sncRNAs. Accordingly, it has been recently shown that the suppressor of RNAi P19 from tomato bushy stunt virus, known to bind and sequester sncRNAs including miRNA, enhances HIV-1 replication [55]. Additionally, the RNAi suppressor function of HIV-1 Tat [56] could be complemented by VP35 from Ebola virus [57] and the NS3 protein of rice hoja blanca virus through sequesteration of small non-coding RNAs [58]. HIV-1 mRNAs associated with RISC are sequestered in the non-polysomal fraction, thereby preventing translation. In agreement with two previous reports [19,21,22], we show that knockdown of RCK/p54, a protein required for miRNA-mediated silencing, led to virus reactivation from PBMCs isolated from HIV-1 infected patients who were undergoing suppressive HAART.

A challenge in AIDS treatment is the need to activate latent viral reservoirs in order to eradicate these viruses through HAART. In this respect, targeting the miRNA processing pathway could offer a strategy that could be exploited to activate latent viral reservoirs, for instance, during HAART. Several molecules have been used to reactivate viral reservoirs [59]. However, none of these approaches provides the sequence specific targeting that can be achieved using siRNA. Recent data suggest that siRNA can be used therapeutically *in vivo *in certain mouse disease models [60] and more recently in non-human primates [61,62]. It remains to be explored whether, as suggested here, the *in vivo *targeting of miRNA-effectors using siRNA can assist in activating latent HIV-1 reservoirs for eradication by HAART.

## Methods

### Constructs

HIV-1 molecular clone pNL4-3Δvif and expression plasmids for APOBEC3G were gift from Olivier Schwartz (Pasteur, France). APOBEC3G H65R/H257R mutant was previously described [63]. HIV-1 vector containing MS2 binding sites and MS2-GFP expression plasmids [64,65] were gift from Alessandro Marcello (ICGEB. Trieste, Italy) and Edouard Bertrand (IGMM. Montpellier, France)

### Transfections

PBMCs were transfected with siRNA or miRNA using the Nucleofector II Device with the appropriate Nucleofection solution according to the manufacturer's instructions (Amaxa). siRNA corresponding to DGCR8 (5'-CAUCGGACAAGAGUGUGAU(dTdT)-3'), Drosha (5'-CGAGUAGGCUUCGUGACUU(dTdT)-3'), RCK/p54 (5'-GCAGAAACCCUAUGAGAUUUU(dTdT)-3'), LSm-1 (5'-GUGACAUCCUGCCACCUCACUU(dTdT)-3'), GW182 (5'-UAGCGGACCAGACAUUUCU(dTdT)-3'), XRN1 (5'-AGA UGA ACU UAC CGU AGA A(dTdT)-3') and CDK9 (5'-CCAAAGCUUCCCCCUAUAATT(dTdT)-3') were synthesized (MWG). Expression level of knock down proteins was analyzed by Western blotting 48 hours after transfection. Briefly, cell-extracts were resolved on SDS-PAGE gels. Proteins were transferred to PVDF membrane by semi-dry electroblotting and probed overnight at 4°C with the primary antibody (anti-Drosha, LSm1, GW182 (Abcam), DGCR8 (Proteintech Group), anti-RCK/p54 (Bethyl)or anti-CDK9 (Santa Cruz), washed and incubated with the appropriate secondary antibody (Amersham) for 1 hour. Proteins were visualized by chemiluminescence according to the manufacturer's protocol (Pierce).

### PBMC isolation and co-culture assay for virus production

Peripheral blood mononuclear cells of HIV-1 infected patients were isolated by lymphocyte separation medium density centrifugation (Lonza). PBMCs from healthy donors were pre-activated using 5 μg/ml PHA (phytohemagglutinin-P, DIFCO)/10 U/ml IL-2 (interleukin-2, Roche) for 72 hours. They were then washed once with PBS and once with RPMI medium before co-culture assay. siRNA transfected HIV-infected PBMC (10^6 ^cells/ml) were co-cultured with pre-activated PBMC (10^6 ^cells/ml) from the same healthy donor in the presence of 10 U/ml IL-2. The culture medium was collected every 3 or 4 days. Fresh pre-activated healthy PBMCs were added to the culture every 7 days. Viral production was measured by quantifying the amounts of p24 in the culture medium using an ELISA kit (Ingen).

### Pseudotyped virion production and single-round infections

The plasmid pNL4-3-env^-^Luc^+ ^harboring a *luciferase *gene (obtained from the NIAID AIDS Reagent Program) was co-transfected with the envelope plasmid pMD.2G encoding the G protein of vesicular stomatitis virus (VSV.G) into human embryonic kidney cells-293T. The virions, named HIV-1VSV-Luc, were collected and filtered using 0.45 μm filters 48 hours post-transfection. HeLa or HeLa CD4+ cells were infected over-night at 37°C, washed and resuspended in DMEM containing 10% FCS. Virus production was monitored in culture supernatant by measuring p24 antigen (Ingen) and by following luciferase activity according to the manufacturer's instructions (Promega).

### Cytoplasmic extracts analysis on sucrose gradients

To isolate cytoplasmic extracts, cells were lysed for 10 minutes in buffer B (5 mM Tris-HCl pH 7.4, 1.5 mM KCl, 2.5 mM MgCl_2_, 0.5% NP40 and protease inhibitor). Nuclei were pelleted by centrifugation for 10 minutes at 10,000 rpm. 2 mg of cytoplasmic extracts were loaded on a 7–47% sucrose gradient. Briefly, 5 layers of 7 to 47% sucrose were prepared in sucrose buffer (20 mM Tris-HCl pH7.4, 80 mM NaCl, 5 mM MgCl_2_, 1 mM DTT and protease inhibitors) and diffused at 4°C for 16 hours to obtain a linear sucrose gradient. 2 mg of cytoplasmic extracts were loaded on the top of the column, and centrifuged for 3 hours at 36,000 rpm in a SW41Ti rotor. After ultracentrifugation, 28 fractions were collected and OD at 254 nm was measured in each fraction using a Nanodrop apparatus (Labtech).

### RNA immunoprecipitation

293 cells were grown in 60 mm dishes and transfected with the indicated plasmids using calcium-phosphate. Cells were harvested 48 hours after transfection, lysed for 15 minutes in RIP buffer (20 mM Hepes, pH 7.5, 150 mM NaCl, 2.5 mM MgCl_2 _× 6H_2_O, 250 mM sucrose, 0.05% NP40, 0.5% Triton X-100) containing RNASIN (Promega) and 1 mM DTT, and centrifuged to pellet debris. Supernatants were incubated overnight with mouse anti-Myc mAb 9E10 (Amersham) at 4°C followed by 2 hours incubation with protein G-Sepharose. Immunoprecipitates were washed with RIP buffer, and nucleic acids were extracted with phenol/chloroform/isoamyl alcohol, isopropanol-precipitated, ethanol-washed and resuspended in RNase-free water. Total RNA was DNase I treated and reverse-transcribed using SuperScript First-Strand Synthesis System for RT-PCR (Invitrogen). RT products were PCR-amplified using either GAPDH (GAPDH forward: GTA TTG GGC GCC TGG TCA CC; reverse: CGC TCC TGG AAG ATG GTG ATG G), HIV-1 (HIV-1 forward: TAG TGT GTG CCC GTC TGT T; reverse: CTC TGG TTT CCC TTT CGC TTT C or Gag-reverse: GAT GGT TGT AGC TGT CCC AG for unspliced HIV RNA), or HDM2 specific oligonucleotides (HDM2 forward: GTA CCT GAG TCC GAT GAT TCC; reverse: ACC TAC TGA TGG TGC TGT AAC). PCR products were resolved on 1.5% agarose/TAE gels containing ethidium bromide. *In vivo *splicing assay and oligonucleotides BSS and SJ4.7A have been described [66]

## Competing interests

The authors declare that they have no competing interest.

## Authors' contributions

CBC, OL and LD carried out most experiments. TR initiated and performed the experiments shown in figures 3 and 4. AZ participated in experiments analyzing the involvement of APOBEC3. WA, JJM, RJ, LY, and SA participated in some of the experiments such as isolation of PBMCs from HIV-1 infected patients. BY and BM directed, supervised and wrote the manuscript.
